# Ultrasonic tissue characterization of vulnerable carotid plaque: correlation between videodensitometric method and histological examination

**DOI:** 10.1186/1476-7120-4-32

**Published:** 2006-08-17

**Authors:** Liz Andréa V Baroncini, Antonio Pazin Filho, Luiz O Murta Junior, Antonio R Martins, Simone G Ramos, Jesualdo Cherri, Carlos E Piccinato

**Affiliations:** 1 Department of Internal Medicine – Faculdade de Medicina de Ribeirão Preto, University of São Paulo, São Paulo, Brazil; 2 Department of Physics and Math – Faculdade de Filosofia, Ciências e Letras de Ribeirão Preto, University of São Paulo, São Paulo, Brazil; 3 Department of Pharmacology – Faculdade de Medicina de Ribeirão Preto, University of São Paulo, São Paulo, Brazil; 4 Department of Pathology – Faculdade de Medicina de Ribeirão Preto, University of São Paulo, São Paulo, Brazil; 5 Department of Surgery and Anatomy – Faculdade de Medicina de Ribeirão Preto, University of São Paulo, São Paulo, Brazil

## Abstract

**Background:**

To establish the correlation between quantitative analysis based on B-mode ultrasound images of vulnerable carotid plaque and histological examination of the surgically removed plaque, on the basis of a videodensitometric digital texture characterization.

**Methods:**

Twenty-five patients (18 males, mean age 67 ± 6.9 years) admitted for carotid endarterectomy for extracranial high-grade internal carotid artery stenosis (≥ 70% luminal narrowing) underwent to quantitative ultrasonic tissue characterization of carotid plaque before surgery. A computer software (Carotid Plaque Analysis Software) was developed to perform the videodensitometric analysis. The patients were divided into 2 groups according to symptomatology (group I, 15 symptomatic patients; and group II, 10 patients asymptomatic). Tissue specimens were analysed for lipid, fibromuscular tissue and calcium.

**Results:**

The first order statistic parameter mean gray level was able to distinguish the groups I and II (*p *= 0.04). The second order parameter energy also was able to distinguish the groups (*p *= 0,02). A histological correlation showed a tendency of mean gray level to have progressively greater values from specimens with < 50% to >75% of fibrosis.

**Conclusion:**

Videodensitometric computer analysis of scan images may be used to identify vulnerable and potentially unstable lipid-rich carotid plaques, which are less echogenic in density than stable or asymptomatic, more densely fibrotic plaques.

## Background

Carotid artery atherosclerosis is responsible for 20% to 30% of ischemic strokes. Several large randomized multicenter trials [1-11] have demonstrated the benefit of carotid endarterectomy (CEA) and recently with carotid artery stenting (CAS) [11-13] in the prevention of stroke, in both symptomatic and asymptomatic disease. In these studies, the degree of internal carotid artery stenosis was the only criterion for selection of patients at high risk for stroke. However, these trials also noted that most patients with high-grade stenosis (>70%) remained stroke free even with medical therapy alone [3]. Factors in addition to the degree of stenosis, such as the histological composition of the plaque, may be responsible for the determination of stroke risk. The composition of plaques from patients with symptoms is significantly different from that of plaques from those without [14-23]. The former contain more total lipid and cholesterol, and less collagen and calcium. Plaque echogenicity as assessed by B-mode ultrasound has been found to reliably predict the content of soft tissue and the amount of calcification in carotid plaques. Fibrous plaques have a highly echogenic quality and the presence of calcium provides a markedly hyperechoic image with shadowing formation. As the lipid content of the plaque increases, the plaque becomes more echolucent [14,24,25]. Nevertheless, the subjective visual analysis of echogenicity provides only a qualitative classification, which can be difficult to reproduce [26]. The present study was designed to establish the correlation between quantitative analysis of ultrasound B-mode images of vulnerable carotid plaque on the basis of a videodensitometric digital texture characterization. and histological examination of the surgically removed plaque.

## Methods

### A. Patients

Thirty-six nonconsecutive surgical inpatients admitted for carotid endarterectomy for extracranial high-grade (≥ 70%) internal carotid artery stenosis were entered into this study between February 2003 and July 2005 from 3 participating hospitals. Local ethical committee approval was obtained for the study and procurement of specimens. Written informed consent was obtained from all patients before each examination. Exclusion criteria were: a disorder that could seriously complicate surgery (3 patients); terminal cancer (1 patient); patient refusal of operation (1 patient); suboptimal ultrasonographic visualization of the atherosclerotic plaque contour/border (1 patient); and surgical specimen inadequate to histological and immunocytochemical analysis (5 patients). The study was conducted on 25 common or internal carotid artery plaques from the 25 remaining patients (18 men and 7 women; mean age 67 ± 6.9 years). A clinical examination, including neurological exam, with particular care taken to establish the number and duration of ischemic events, and a record of the time from the last symptom and the operation, was obtained from each patient. Before surgery, all patients underwent a: 1 – either cerebral angiography or magnetic resonance angiography and Duplex ultrasound for grading carotid artery stenosis and assessment of intracranial arterial system; and 2 – either computer tomography (CT) or magnetic resonance brain scan. The presence or absence of infarction in the corresponding middle cerebral artery territory was noted. Focal cerebral ischemic events were defined as transient ischemic attack (TIA), amaurosis fugax (AF), central retinal artery occlusion (CRAO), or cerebrovascular accident. Patients were considered to be symptomatic if they had experienced AF, TIA or stroke ipsilateral to the carotid lesion being studied. Silent infarcts and lacunar symptomatology, diagnosed by a neurologist based on clinical and brain computer tomography (CT) scan and/or magnetic resonance imaging (MRI) located ipsilateral to the stenosis, were also considered symptomatic. On the other hand, patients without any history of recent neurologic symptoms or with nonspecific, nonhemispheric symptoms such as dizziness and vertigo were considered asymptomatic. Each patient was then assigned preoperatively to 1 of 2 groups on the basis of their symptoms: group I symptomatic patients (n = 15; mean age 67.4 ± 6.4 years) and group II (n = 10; mean age 65.2 ± 7.9 years) consisting of all asymptomatic patients. At the baseline examination, measurements of height, weight, body mass index, blood pressure, fasting serum total cholesterol, HDL cholesterol, LDL cholesterol, triglycerides, fasting plasma glucose, electrocardiograms and information about coronary artery disease, diabetes mellitus and smoking habits was collected. Percentages of carotid diameter reduction, procedural methods, concomitant therapy, age, sex, and risk factors did not differ between the 2 groups (Table 1).

**Table 1:** Patient's Characteristics

	Group I (n = 15)	Group II (n = 10)
Age, years	67.4 ± 6.4	65.2 ± 7.9
Sex, M/F	11/4	7/3
Hypertension	10	1
Diabetes mellitus	2	3
Active Smoking	3	3
Hypercholesterolemia	2	1
CAD	4	0
Aspirin	15	10
Statin	5	4
ACE inhibitors	9	8
Ticlopidine	4	1

CAD = coronary artery disease

### B. Ultrasonographic image acquisition and preprocessing

The patients underwent carotid endarterectomy 1 to 2 days after ultrasound assessment. Conventional echo images were acquired with a commercially available 2D ultrasonic imaging system (Hewlett-Packard Sonos 5500, Andover, Massachusetts). The system characterized arterial tissue at the bedside using a 5- to 12- MHz multifrequency linear transducer for all studies. This software enables the acquisition; storing and retrieving of a sequence of continuous 2D conventional images, forming a continuous loop digital recording of 2 s (60 frames in 2 s). Anterior, lateral and posterior projections were used to image the plaque longitudinally. The position of the probe was adjusted so that the ultrasonic beam was vertical to the artery wall. Offline analysis of the 2D images was performed by retrieving the previously stored data from the built-in optical disc drive in the system. For videodensitometric analysis, the images from the magnetical optical disk were loaded into a computer where a specific software program (CaPAS – Carotid Plaque Analysis Software) was designed. Selection was done, such that plaque contour/border, area, and contrast were optimized, subjectively judged. Only the image plaque at anterior vessel wall was analysed. The selected static frames considered appropriate for analysis, should fulfil these criteria: 1) the blood, in the vicinity of the plaques, was dark and echoically uniform, and 2) the atherosclerotic plaque was well delineated, horizontal, and with maximum thickness.

### C. Quantitative texture analysis

All plaque images were evaluated by the software CaPAS for texture parameters including a set of first-order (mean gray level; and standard deviation) and of second-order (entropy, energy, and homogeneity) parameters. The mean gray level (MGL) represents the median of the frequency distribution of gray tones of the pixels included in the region of interest (gray scale median of the region) in a scale of 256 gray tones (0 = darkest tone; 255 = brightest tone) [25]. Dark (hypoechoic) regions were associated with a gray scale median (GSM) that tends to approach 0, whereas bright (hyperechoic) regions were associated with a GSM that tended to approach 255. The standard deviation (SD) is an expression of the spreading of the distribution from the mean value, i.e., the overall contrast. The energy or angular second-moment value increases when the co-occurrence matrix elements are very unequal. Entropy and homogeneity reflects the coarseness of the image, as its value increases when homogeneity is reduced, i.e., when co-occurrence matrix elements tend to be equal and the diagonal concentration lowers. The mathematical definitions of these texture parameters are described in previous articles [27]. All plaque images were normalized by using two echo-anatomic points: the gray scale median (GSM) of the blood and the GSM of the periadventitia region. After normalization, each image plaque was outlined manually three times by the same examiner in its longitudinal section. Mean score of these three sequential measurements was used as a final value.

### D. Procurement of tissue specimens and histological analysis

Carotid plaques were obtained immediately after endarterectomy. All surgeries were performed with standard surgical techniques, and with minimal manipulation of the specimen. No attempts were made to evaluate the presence and the degree of surface ulceration or thrombus. The plaque should be removed in bloc, without fragmentation or significant distortion. After removal, the section of plaque for histological analysis was placed in fresh 4% paraformaldehyde solution and partly decalcified overnight, in order to be sectioned subsequently. The samples were transected transversely at 3 to 4 mm, and embedded in paraffin. For the most of the specimens, five to six blocks were avaiable. Histological analysis was performed by an experience pathologist (SGR) who was unaware to the ultrasound results. Tissue specimens were analysed for lipid, fibromuscular tissue and calcium and expressed as the percentage of the total plaque area obtained.

### Statistical analysis

Categorical variables were expressed as percentages and continuous variables were expressed as mean ± SD (median). The comparison of the histological and videodensitometric parameters among the groups was done by non-parametric test of Wilcoxon rank-sum test or Chi-square test as appropriate and the correlation between histological and videodensitometric data was done by the non-parametric Spearman test. Statistical significance was indicated by a value of *P *< 0.05. The intra and inter-examiner variability in ultrasonographic measurements was tested in all carotid images as proposed by Lin [28].

## Results

### A. Histological examination

Tissue specimens were analysed for lipid, fibromuscular tissue and calcium. The percentage of these three tissue components was determined in each plaque section. The percentage of fibromuscular tissue had higher prevalence in the group II (75,86 ± 11,46) and lower prevalence in the group I (51,87 ± 13,01) with statistic significance (*p *= 0.04). The percent of lipid tissue had the opposite results (40,10 ± 15,80 for group I and 19,57 ± 9,96 for group II; *p *= 0,05). The percentage of calcium did not differ between the two groups (Table 2).

**Table 2:** Histological and videodensitometric parameters according clinical groups (mean ± sd).

		I (15)	II (10)	*p*
Histological Parameters	Fibromuscular tissue (%)	60,36 ± 5,410	75,86 ± 3,623	0,0445
	Lipid (%)	32,32 ± 4,705	19,57 ± 3,151	0,0560
	Calcification (%)	7,313 ± 1,990	4,578 ± 2,301	0,3835
Videodensitometric Parameters	Mean Gray Level	0,389 ± 0,033	0,565 ± 0,0405	0,003
	Standard Deviation	3,774 ± 0,429	4,985 ± 0,399	0,063
	Entropy	5,66 ± 0,1036	5,580 ± 0,1371	0,618
	Energy	0,006 ± 0,0009	0,023 ± 0,008	0,025
	Homogeneity	0,201 ± 0,0115	0,233 ± 0,002	0,1689

### B. Videodensitometric analysis

Among first-order parameters, the MGL was effective in distinguishing group I versus group II (*p *= 0.04), showing significant lower values in group I. The standard deviation had the same behaviour but without statistic significance. Among second order parameters, energy distinguished groups I and II with lower values in group I (*p *= 0.02). Homogeneity and entropy did not find any significant difference (Table 2).

### C. Correlation between histological examination and videodensitometric analysis

**Figure 1:**
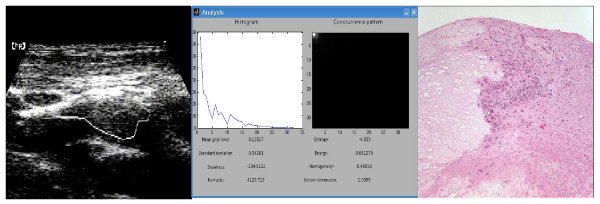
Vulnerable carotid plaque: Left Panel – Ultrasound B-mode image of a hypoechoic and homogeneous plaque with the region of interest (ROI) delimiting the area to be analysed in Mid Panel; Mid Panel – Videodensitometric analysis showing the histogram and cooccurence matrix distribution along with continuous variables generated by CaPAS; Right Panel – Histological analysis: great necrosis area, lipid tissue and inflammatory infiltrate(dotted pink area).

**Figure 2:**
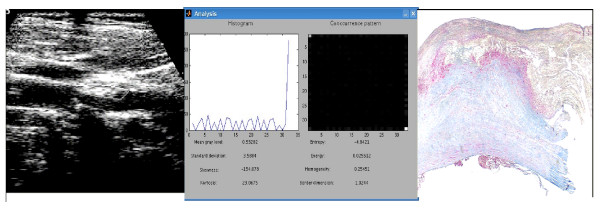
Stable carotid plaque: Left Panel – Ultrasound B-mode image of a hyperechoic and heterogeneous plaque with the region of interest (ROI) delimiting the area to be analysed in Mid Panel; Mid Panel – Videodensitometric analysis showing the histogram and cooccurence matrix distribution along with continuous variables generated by CaPAS; Right Panel – Histological analysis: great area of fibrosis (blue area).

**Figure 3:**
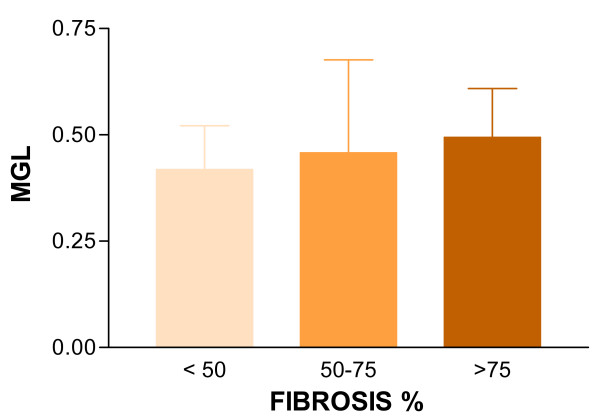
Mean Gray Level (MGL) and proportion of Fibrous Tissue.

There was no statistical significant correlation between the histological examination and videodensitometric parameters. However, considering MGL and proportion of fibrous tissue, progressively greater values was found from specimens with < 50% to >75% of fibrosis. Figures 1 and 2 show examples of ultrasound B-mode images, CaPAS parameters and histological analysis. Figure 3 shows MGL values related to proportion of fibrous tissue.

### D. Intra and inter-examiner variability

The inter-examiner variability acquired good agreement between the measurements. The intra-examiner variability found concordance for first order videodensitometric parameters and for entropy, but not for energy and homogeneity. The mean inter-examiner variability ranged: for MGL from 0,1607 to 0,9622; for SD from 0,3236 to 0,9809; for entropy from 0,5406 to 0,9459; for energy from 0,1811 to 0,6981; and for homogeneity from 0,3244 to 0,9049. The mean intra-examiner variability ranged: for MGL from 0,4280 to 0,8150; for SD from 0,3958 to 0,7996; for entropy from 0,1085 to 0,6141; for energy from - 0,0342 to 0,4546; and for homogeneity from - 0,1181 to 0,2073.

## Discussion

### Comparison with previous studies

Mazzone et al [27] selected 47 images of carotid plaques from 10 patients and correlated visual assessment of plaque echodensity and homogeneity with the results obtained by mathematical descriptors of tissue texture. The plaques were first assigned as soft, fibrotic and calcific according visual approach. In the first-order parameters of tissue texture, the mean gray-level was significantly lower in soft compared with fibrotic and calcific plaques, whereas the remaining first-order parameters (Standard deviation, Skewness and Kurtosis) overlapped in the three groups. In the evaluation of plaque homogeneity, second -order parameter entropy could clearly separate homogeneous and dishomogeneous plaques. Beletsky et al [29] performed densitometric analysis of B-mode images of carotid plaques in nine patients and compared with histological examination. Plaque components were grouped as follows: soft plaque/organized thrombus, intraplaque haemorrhage/fatty deposition, fibrosis, and densely calcified plaque. They found soft plaque/organized thrombus had a lower density than intraplaque haemorrhage/fatty deposition, which in turn had a lower density than fibrosis. Calcified plaque had the highest density measurement. Wilhjelm et al [5] compared subjective classification of the ultrasound images with 16 first- and 7 second-order statistical features extracted from regions of the plaque in still ultrasound images and with histological analysis of the surgically removed plaque in 52 patients. All patients had experienced ipsilateral neurological symptoms. This study obtained good accuracy between visual subjective and densitometric evaluation, but no agreement was found with histological analysis. They failed in prediction of soft and calcified materials. Sayed Aly and Christopher C. Bishop [30] used mean pixel value (MPV) of ultrasound images to assess the level of echogenicity and compared with histological findings in carotid plaques of 17 patients. Tissues of known type in a human volunteer were examined (blood, fat, muscle, and fibrous and calcified tissues). The MPV of the pixels in the tissue of interest in the image was used as the parameter to identify the echogenicity of the structure. The histological study was designed to test whether the findings in the previous study could be extended to the assessment of the morphology of atheromatous plaques. The findings of computer-assisted gray-scale image analysis of these specimens have been verified by the histological findings, and a good correlation has been shown. This study has shown that as the soft (fat and blood) content of the plaque increased, the MPV decreased, and as the fibrocalcific tissue content of the plaque increased, the MPV increased. This relation between the MPV and plaque histology has been found to be significant (*p *< 0.002). Brajesh K. Lal et al [31] in a similar study compared pixel distribution analysis (PDA) of B-mode ultrasound images with histologic features of atherosclerotic carotid plaques in two groups of patients: 13 asymptomatic and 7 symptomatic (13 weeks mean time before surgery). The authors found significant different amount of intraplaque haemorrhage, fibromuscular tissue, and calcium between two groups with good correlation with PDA. Recently Sztayzel et al [32] in 28 patients (13 symptomatic, mean time of 4 weeks before surgery) correlated the mean gray level with histological findings. The plaque pixels were mapped into 3 different colours, namely red, yellow, and green, depending on their gray-scale value. Thresholds were chosen as: lowest gray-scale values (<50 mapped in red), intermediate values (between 50 and 80 mapped in yellow), and highest values (>80 mapped in green). They determined for each plaque the predominant colour present on the surface, which was defined as the upper third part of the lesion, and the predominant colour of the whole plaque or plaque segment. Fibrosis, haemorrhage, calcification, or necrotic/lipid core were respectively expressed as large or small if they occupied >50% or <50% of the total area of the plaque. They found a good correlation with histological findings and also allowed identification of some characteristics like the thickness of fibrous cap and the juxtalumenal position of the necrotic core.

Clearly, all these studies identify the MGL as the first order parameter able to differentiate a predominant tissue component (lipid, fibromuscular tissue and calcium). In the present study a linear association between MGL and fibromuscular tissue could be found, even though there was only a tendency to the other first order parameter. Classically, the first order parameters correlate with structural tissue components based on attenuation of ultrasound beam and reflect the image bright intensity without determine regional variations inside the plaque. The high variability found could be explained in part by the lack of any corrections to the machine settings among patients. The second order parameters are associated with texture pattern, depending on less of the bright and more of the image heterogeneity. In the present study, considering the second order parameters, Energy was able to distinguish the groups I and II (lower values in group I) but no correlation could be made with quantitative histological structural components. These findings are interesting and not incorrect, as second order parameters do not reflect the amount of specific tissue component but its arrangement and spatial organization. They could be assessing the tissue heterogeneity or a disarrangement caused for example by an active inflammatory process not considered in the present study. The atherosclerotic plaque is not a one-way progression from a "soft" to a "hard" plaque. It is a continuous disease where the plaque constantly suffers from reparative process during the evolution of atherosclerosis and is further supportive.

### Study limitations

Some limitations of our data could be responsible for the fact that the agreement between histological and videodensitometric findings was not higher. First, the small number of patients was an important study limitation. This limitation will not be easily overcomed, since the improvement of carotid artery stenting techniques will make histological analysis of carotid plaques an infrequent procedure. Second, by necessity, only a small proportion of each plaque was examined microscopically, and it may well be that features were missed in some patients. Also, histological separation between calcium and lipid tissue is very difficult, as calcification could be either a delimited area or be mixed with the overall lipid tissue. Third, only plaque images at anterior wall of vessel were considered for analysis and dark regions due to the shadowing effects of calcium material were discharged. Finally, the software CaPAS used in this study needs improvement, mainly regard with second order statistics parameters that did not find good concordance between intra-examiner variability. The numbers are disposed in decimals and any change will make a great difference.

## Conclusion

Videodensitometric computer analysis of scan images may be used to identify vulnerable and potentially unstable lipid-rich carotid plaques, which are less echogenic in density than stable or asymptomatic, more densely fibrotic plaques.

## Competing interests

The author(s) declare that they have no competing interests.

## Authors' contributions

LAVB designed the study and carried out the ultrasound evaluations of carotid plaques.

APF also designed the study and made the statistical analysis.

LOMJ created and adapted the CaPAS software used in this study.

ARM also designed the study and oriented in the histologic analysis of the plaques.

SGR made the histological examination of the carotid plaques.

JC and CEP are vascular surgeons that made possible to obtain the carotid plaques.

All authors read and approved the final manuscript.
